# Effect of Cooling Rate on Morphology of TiAl_3_ Particles in Al–4Ti Master Alloy

**DOI:** 10.3390/ma10030238

**Published:** 2017-02-27

**Authors:** Jianhua Zhao, Tao Wang, Jing Chen, Lu Fu, Jiansheng He

**Affiliations:** 1 State Key Laboratory of Mechanical Transmission, College of Materials Science and Engineering, Chongqing University, Chongqing 400044, China; 20113869@cqu.edu.cn (T.W.); 20150902010@cqu.edu.cn (J.C.); 20113754@cqu.edu.cn (L.F.); jiansheng.he@cqu.edu.cn (J.H.); 2 National Engineering Research Center for Magnesium Alloys, Chongqing University, Chongqing 400044, China

**Keywords:** Al–4Ti master alloy, cooling rate, morphology evolution, microstructure, supersaturation

## Abstract

The Al–4Ti master alloy was fabricated by aluminum (Al) and sponge titanium particle in a resistance furnace at different cooling rates. This work aims to investigate the relationship between the cooling rate and morphology of TiAl_3_. The microstructure and composition of master alloys at different cooling rates were characterized and analyzed by optical microscopy (OM), X-ray diffraction (XRD), differential scanning calorimetry (DSC), and SEM with energy dispersive spectroscopy (EDS). The results showed that various morphologies of TiAl_3_ particles in the Al–4Ti master alloy could be acquired at different cooling rates. Petal-like, blocky, and flake-like TiAl_3_ particles in the Al–4Ti master alloy were respectively acquired at the cooling rates of 3.36 K/s, 2.57 K/s, and 0.31 K/s. It was also found that the morphology of TiAl_3_ particles in the prepared master alloy changed from petal-like to blocky, then finally to flake-like, with the decrease of cooling rate. In addition, the morphology of the TiAl_3_ particles has no effect on the phase inversion temperature of Al–4Ti master alloy.

## 1. Introduction

Grain refinement plays a vital role in the production of aluminum (Al) alloy. It is important to control the grain size (which is beneficial for improving the surface quality), decrease the hot tearing tendency, and reduce the mold-filling time. As reported, the fine and equiaxed grain structures have greatly improved mechanical properties, such as toughness and yield strength. What is more, finer grain is advantageous in that it improves the casting speed and the homogeneity of cast structures [1,2,3]. In recent years, the refined Al alloys are widely used in aerospace, national defense, and automobile industries. For this reason, projects involved in the study of grain refinement have become significant development projects [4]. As we know, the addition of Al–Ti, Al–B, Al–Ti–B, and Al–Ti–C, as well as recently developed master alloys with other third elements (e.g., Zn) [5,6,7] has become a good way to decrease the mean grain size of the inoculated metals and alloys. The grain refinement performance of Al–7Ti master alloy in Al–7Si alloys has been observed by Virupaxi [8]. Yucel [9] showed that AlB_3_ can contribute to the average grain size of AlSi10Mg and AlSi12Cu. Ghadimi et al. [3] indicated that the smaller the size of particles in Al–5Ti–B master alloy are, the better the refining performance is. Although Al–Ti alloys are easy to overlook because of their lower grain refinement efficiency (compared with Al–Ti–B master alloy), Al–Ti alloys have a huge potential in certain fields such as aluminum foil and electronic accessories [10].

Many experimental results have been reported, which have described the influence of the manufacturing process parameters on the microstructure of the master alloys, including reaction temperature, time, and composition. Lee et al. [11] forecasted that the structures of TiAl_3_ particle are influenced by the cooling rate of the alloy. Kandalova et al. [12] elucidated that Al–Ti master alloys showed the hereditary effect of the initial ingot grain size. TiAl_3_ crystals with different sizes, morphologies, and quantities would affect the pattern of the grain-refining properties with the holding time. In the present investigation, a detailed study was conducted to determine the influence of various process parameters—such as thermomechanical treatments [3], sequence of salt addition [13], reaction temperature and time [8,14], and stirring conditions [15]—on the microstructure of the master alloys. However, few studies have touched on observations of the detailed evolution of the different morphologies at different cooling rates of TiAl_3_ particles in Al–Ti master alloy. With this backdrop, it is necessary for us to study the different morphologies of TiAl_3_ particles in each evolution phase and their formation mechanism of Al–4Ti alloy at different cooling rates.

## 2. Experimental

### 2.1. Materials

**Figure 1:**
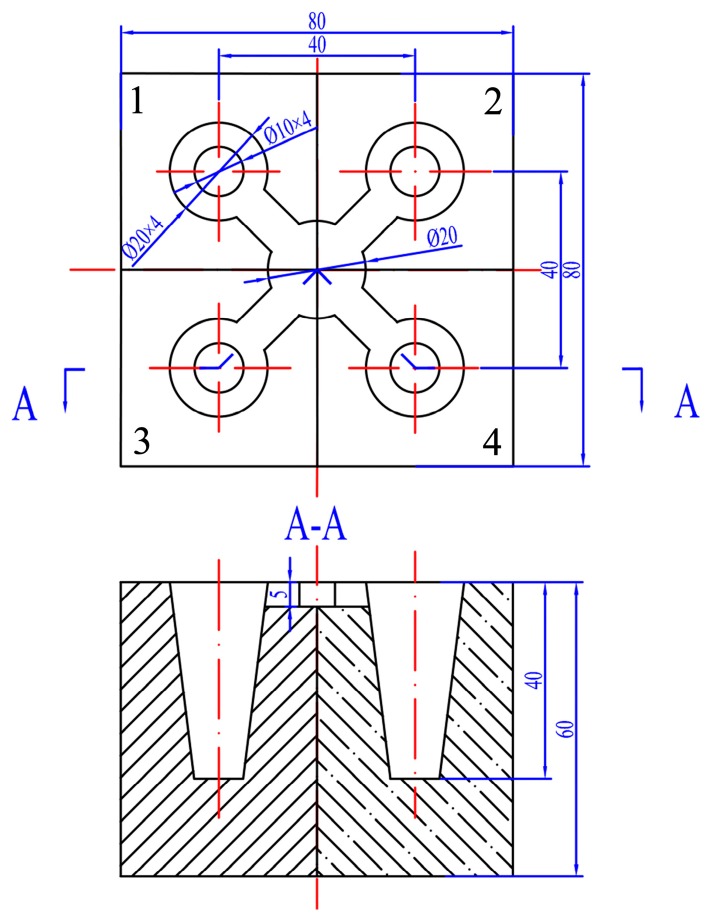
The geometry of the casting model: (**1**) graphite; (**2**) copper; (**3**) steel; (**4**) sand. (Unit: mm).

The geometry of the model, which is made up of four different materials, is shown in Figure 1. The four materials are graphite, copper, steel, and sand. This configuration allowed the acquisition of the unequal solidification rates, which consequently resulted in different microstructures in the process of casting. The common methods used for preparation of Al–Ti alloys are mainly the halide salt reactions of Al and K_2_TiF_6_ and the reaction of Al and titanium sponge particles. In this paper, considering the actual industrial production cost, the Al–4Ti alloy was prepared by the titanium particle method. Pure aluminum ingots, titanium sponge, and modifier were prepared before the experiment. Pure aluminum ingots were melted in a crucible placed in an electrical resistance furnace. As the melt temperature was increasing to 880 °C, the titanium sponge was proportionally added into the melt. Na_3_AlF_6_ was used to improve the dissolution speed of the titanium sponge. After the titanium sponge was melted, the dross was removed from the melt surface. The modifier—whose composition was KCl, NaCl, and K_2_TiF_6_—was added into the melt for 10 min and uniformly agitated. The modifier melt was poured at 870–880 °C into the mold, which was heated to 200 °C beforehand after being degassed with dry hexachloroethane, and joined in the melt for 8 min. In order to obtain the average cooling rate of the melt in the mold with different materials, the AnyCasting simulation software (Version 2.4, AnyCasting Co., Ltd., Korea) was used. As a result, the cooling rates of the mold center were determined as: graphite = 3.36 K/s, copper = 2.57 K/s, steel = 1.65 K/s, and sand = 0.31 K/s.

### 2.2. Microstructural Analysis

After solidification, specimens were cut out from the sections where the distance was 20 mm from the bottom. The metallographic specimens were prepared through mechanical grinding and polishing with high alumina polishing powder. Electrolytic etching with the solution (5 mL HClO_4_ in volume fraction of 70%, 95 mL anhydrous ethanol) and anodization for 12–15 s at a current of 0.2 A (the etching time depended on the size of the section) were performed to reveal the microstructure, as well as the morphology of the Al–Ti compound. Quantitative microstructural analysis was carried out by using an optical microscope (OM). A study of the three-dimensional morphology of particles in the Al–4Ti master alloy was conducted after a deep etching, using a TESCANVEGA II scanning electron microscope (SEM) (TESCAN, Brno, Czech Republic). The etchant contained NaOH and distilled water. The distribution of chemical compositions was measured using an energy dispersive spectroscopy (EDS) detector. The phase constitutions on the fracture surfaces of the specimens were identified by using a D/max2500PC X-ray diffractometer (XRD) (PANalytical B.V., Almelo, The Netherlands). Differential scanning calorimetry (DSC) (NETZSCH, Selb, Germany) analysis was carried out to examine the precipitation temperature of TiAl_3_ particles with different morphologies.

## 3. Results and Discussion

### 3.1. Microstructure of Al–4Ti Master Alloy

**Figure 2:**
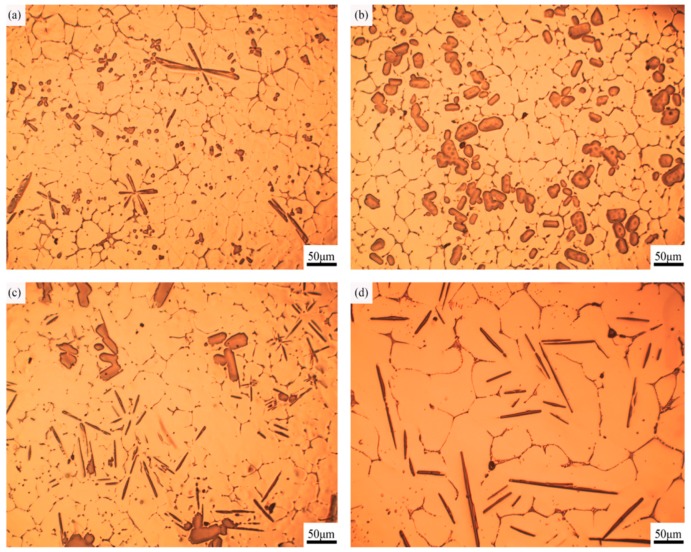
Optical micrographs of Al–4Ti master alloys at different cooling rates: (**a**) 3.36 K/s; (**b**) 2.57 K/s; (**c**) 1.65 K/s; (**d**) 0.31 K/s.

Figure 2 shows the typical optical micrographs of Al–4Ti, which is produced via the reaction between titanium sponge and molten aluminum. It can be observed that an overall uniform dispersion of petal-like TiAl_3_ particulates appearing in the aluminum matrix when Al–4Ti master alloy was cooled down in the graphite mold (Figure 2a). The presence of TiAl_3_ particles is confirmed by XRD and EDS analysis. The average size of the majority of petal-like TiAl_3_ particulates is small. Most of the primary particles are separated because there is not enough time to congregate at a high cooling rate. Figure 2b shows that blocky TiAl_3_ particulates are distributed on the aluminum matrix uniformly when the cooling medium is copper. The second phase was mainly made up of blocky and flake-like TiAl_3_ when the melt was cooled down in the steel mold (Figure 2c). When the melt was cooled in the sand mold, the TiAl_3_ particles are fully converted into long, flake-like morphologies (Figure 2d). At the same time, it is found that the aluminum grains grow up obviously when the time of crystallization is long enough. This can be attributed to the decrease of the cooling rate. Thus, the results clearly suggest that the morphology of TiAl_3_ particles in the prepared master alloys was altering from petal-like to blocky, and finally to flake-like as the cooling rate was decreasing.

**Figure 3:**
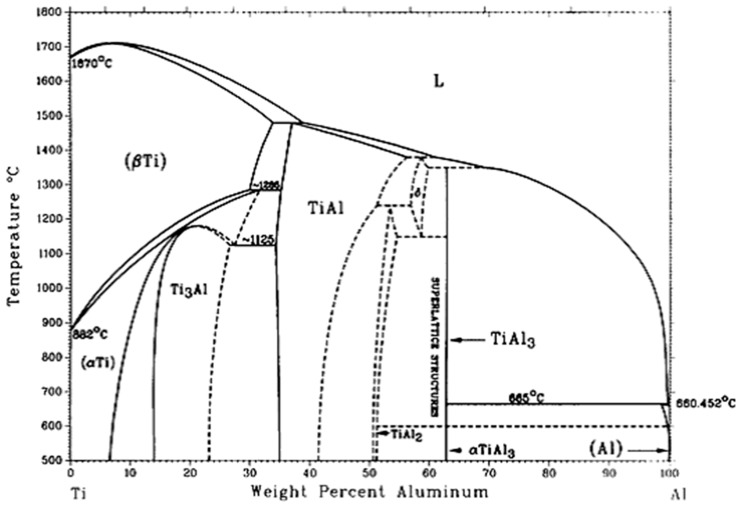
The Al–Ti phase diagram [17].

Figure 2 also reveals that TiAl_3_ particles are within the α-Al grains. There is a possibility that we can infer the basis of this phenomenon from the Al–Ti phase diagram (Figure 3). According to the peritectic reaction at 665 °C, the TiAl_3_ particles act as nucleation sites, and they are surrounded by the molten aluminum. So, it is obvious that the TiAl_3_ particles stay in the center of α-Al grains. The result of TiAl_3_ particles coated by aluminum is similar to that of Wang’s study [16]. In addition, vigorously stirring before pouring is helpful to homogeneous dispersion of the TiAl_3_ particulates. Figure 2b shows that some blocky TiAl_3_ particles have an aggregation tendency. This can be explained by a slight stirring force, which can hardly overcome the force of viscosity and gravity.

**Figure 4:**
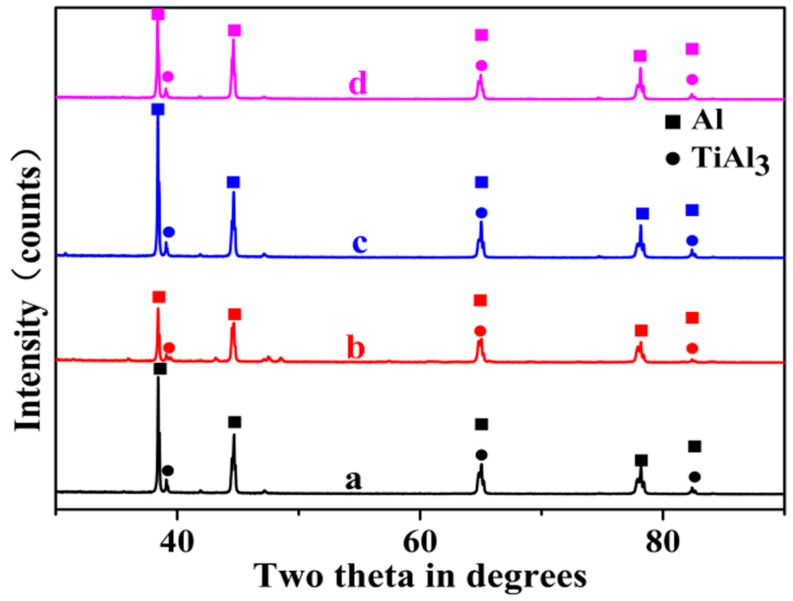
X-ray diffraction (XRD) patterns of Al–4Ti master alloys solidified at different cooling rates: (**a**) 3.36 K/s; (**b**) 2.57 K/s; (**c**) 1.65 K/s; (**d**) 0.31 K/s.

Figure 4 shows the XRD result of Al–4Ti master alloys solidified at different cooling rates. It indicates that all of the prepared Al–4Ti master alloys at different cooling rates consist of Al matrix and TiAl_3_ phase. The phases observed in Al–4Ti master alloy are confirmed by the binary Al–Ti phase diagram. It is obvious that there is no prominent change in the phases.

**Figure 5:**
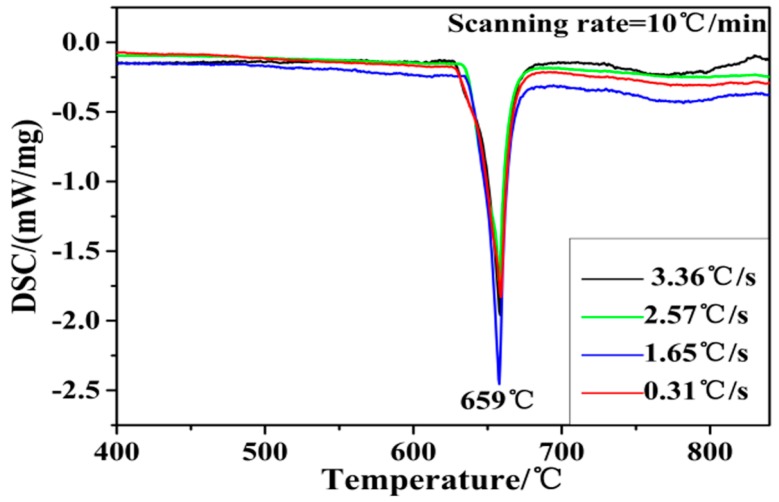
Differential scanning calorimetry (DSC) curve of Al–4Ti master alloy at different cooling rates.

Figure 5 shows a typical DSC trace for melting of Al–4Ti master alloy at different cooling rates, which is scanned at a heating rate of 10 °C/min under a protective argon atmosphere. In the DSC curves, it is very obvious that there is only one endothermic peak at 659 °C in each curve, meaning that the master alloy has melted at 659 °C. Furthermore, the trend of the four curves is basically consistent, and there is no obvious peak at 700–850 °C. This suggests that the specimens produced under different cooling rates have the same phase inversion temperature. Thus, the decrease of the cooling rate and the different morphologies of the TiAl_3_ particles have no obvious effect on the phase inversion temperature of Al–4Ti master alloy.

### 3.2. Morphology of TiAl_3_ Particles in Al-4Ti Master Alloy

Generally, there are several morphologies of TiAl_3_ and various phases in the samples. In this study, there are three different kinds of TiAl_3_ particles in Al–4Ti master alloy, which are interpreted in the following sections.

#### 3.2.1. Petal-Like TiAl_3_ Particles

**Figure 6:**
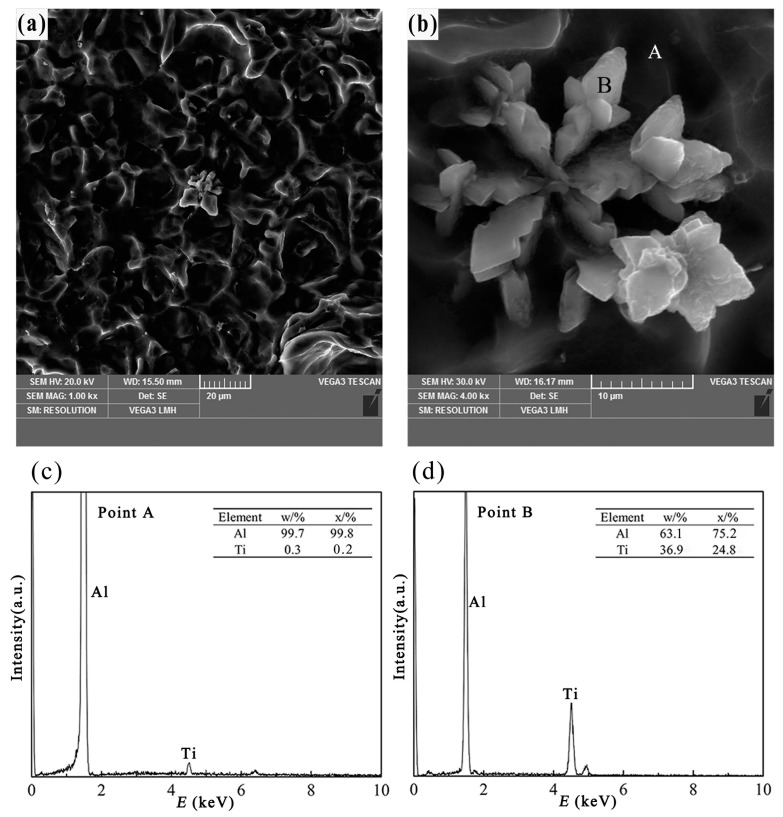
Three-dimensional morphology and point scan elements analysis of petal-like TiAl_3_, (**a**,**b**) three-dimensional morphology of the petal like TiAl_3_ particle; (**c**) EDS spectra of point A; (**d**) EDS spectra of point B.

Figure 6a,b displays the three-dimensional morphology of petal-like TiAl_3_ particles in Al–4Ti master alloys, and the melt was solidified in a graphite mold. When the cooling rate is high (3.36 K/s, at a distance of 20 mm from the bottom), the morphology of the TiAl_3_ particles exhibits a petal shape, which includes several lamellas. It is obvious that some of the petals consist of three or four thick plates, all of which are perpendicular to each other, as shown in Figure 6b. Al matrix and TiAl_3_ particles are found from the point scan elements analysis, as shown in Figure 6c,d, respectively. Because of the high cooling rate, the petal-like TiAl_3_ particles are tiny. Meanwhile, according to the distribution of TiAl_3_ particles in Figure 2, it is clearly shown that the free TiAl_3_ particles in the melt are nucleation centers, so, the lamellas started to grow outward from the center. It is interesting that there is a groove shape (as shown in Figure 6b) in the middle of the petal-like TiAl_3_ particles, which can also be found in the study of Zhao et al. [10]. The petal-like TiAl_3_ particles have been rarely reported and the cause of the formation has not been described clearly yet. However, it can be inferred that the petal-like TiAl_3_ particles have formed in a zone, where the content of Ti is adequate and the cooling rate is high.

If the cooling rate is high (about 3.36 K/s) and the content of Ti is adequate, the petal-like TiAl_3_ particles start to develop. Under this condition, the growth process of TiAl_3_ needs remote diffusion of Ti atoms to meet the desired Ti of edge growth. The velocity of atomic diffusion decreases with the decrease of the melt temperature, and subsequently the growth rate of petal-like particles would decrease due to the lack of Ti atoms. When there are sufficient Ti atoms, it is interesting that the petal-like TiAl_3_ particles do not stop growing until lamellas contact neighboring petal lamella. The lamella of the petal-like TiAl_3_ particle exhibits a composition of several thick plates, which are perpendicular to each other. This is due to the fact that the degree of undercooling in the direction that is perpendicular to the larger plane of the thick-plate TiAl_3_ particle is greater than that of the parallel direction. In addition, the content of Ti is greater than that in the parallel direction of the old thick-plate TiAl_3_ particle in the melt. So, the new thick plate can grow vertical to the old one easily. This growth process of the petal-like TiAl_3_ particle is similar to that of dendrite growth. This phenomenon can be explained by twinning, which was put forward by Arnberg et al. [18]. However, with the increase of the cooling time, the emulsification occurs on the lamellas of petal-like TiAl_3_. After that, the morphology of the particle becomes much more like a passion flower.

#### 3.2.2. Blocky TiAl_3_ Particles

**Figure 7:**
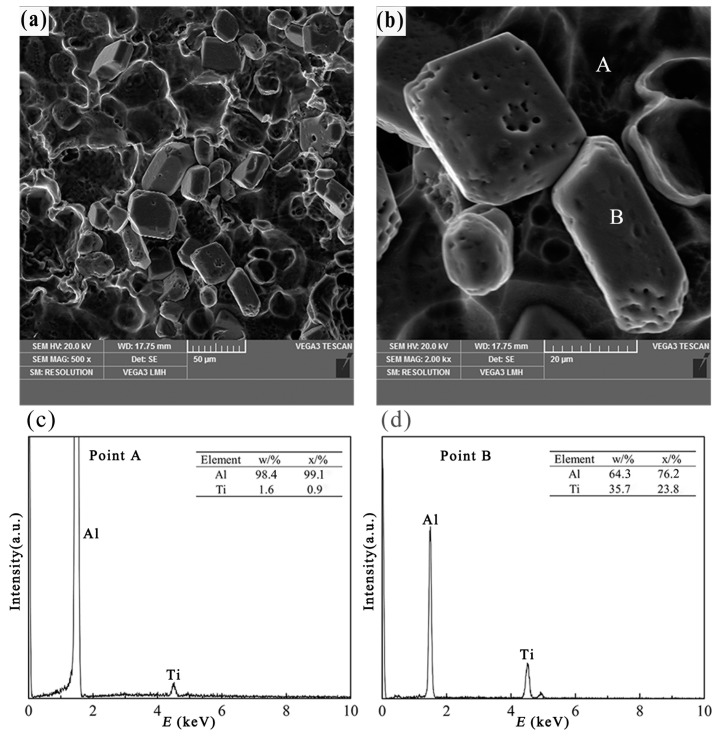
Three-dimensional morphology and point scan elements analysis of block-like TiAl_3_, (**a**,**b**) three-dimensional morphology of the blocky TiAl_3_ particle; (**c**) EDS spectra of point A; (**d**) EDS spectra of point B.

Figure 7a,b reveals the three-dimensional morphology of blocky TiAl_3_ particles in Al–4Ti master alloys, resulted from the melt being solidified in a copper or steel mold. At an intermediate cooling rate (2.57 K/s or 1.65 K/s, at a distance of 20 mm from the bottom), the primary particles exhibit a cubic shape or hexagonal platelet morphology, as shown in Figure 7a,b. Most of the blocky TiAl_3_ particles present regular geometries, but there are still some particles that are nearly spherical. Furthermore, the edges of the blocky TiAl_3_ particles are smooth. This phenomenon can be explained by the dissolving of TiAl_3_ particles. Because of the medium cooling rate, the time spent by the TiAl_3_ particles in the melt could be longer and, thus, the edge and the corner of the blocky TiAl_3_ particles would therefore gradually dissolve. The TiAl_3_ particles were confirmed through EDS, shown in Figure 7c,d. These results appear to coincide with the study of Auradi [8], which described the blocky TiAl_3_ during the preparation of Al–7Ti master alloy. Further, some agglomerated particles of blocky TiAl_3_ can also be observed in Figure 7a. The blocky particulates have high volume/surface area ratio, which was reported by Lee et al. [11]. Therefore, the agglomerated particles can minimize the surface energy of the blocky particles.

When the intermediate cooling rate is about 2.57 K/s, the TiAl_3_ particles are mainly blocky. It can be found in Figure 7b that the blocky TiAl_3_ particles exhibit a regular cubic shape, and the major surfaces of the cubic shape are exposed to the Al melt. This can be confirmed in the study of Arnberg et al. [18], which was reported that the blocky crystals expose {001} and {011} planes to the Al melt, providing the opportunity to simultaneously grow in both <001> and <110> directions. At a medium cooling rate, the degree of supersaturation of Ti atoms in the Al melt is high, and the diffusion rate of Ti atoms is substantially the same in all directions. As a result, the blocky particulates grow in both {001} and {011} planes with supersaturation of Ti atoms in the Al melt. Arnberg et al. [18] also suggested that the planes of (001)TiAl_3_ and (011)TiAl_3_ have a good match with the (021)α-Al and (012)α-Al planes, respectively (i.e., (001)TiAl_3_//(021)α-Al and (011)TiAl_3_//(012)α-Al). In addition, Schumacher et al. [19] also suggested that the {112}TiAl_3_ planes have a good orientation relationship with the {111}α-Al planes. The low lattice misfit structure together with the peritectic reaction provides a great opportunity for the blocky TiAl_3_ particles to stay in the α-Al grain center (Figure 2b). Moreover, because of the tiny diffusion influence of Ti atoms, the preferential growth trend of TiAl_3_ particles is not so obvious that the growth rate of each plane is roughly the same, which results in the formation of blocky TiAl_3_ particles.

#### 3.2.3. Flake-Like TiAl_3_ Particles

**Figure 8:**
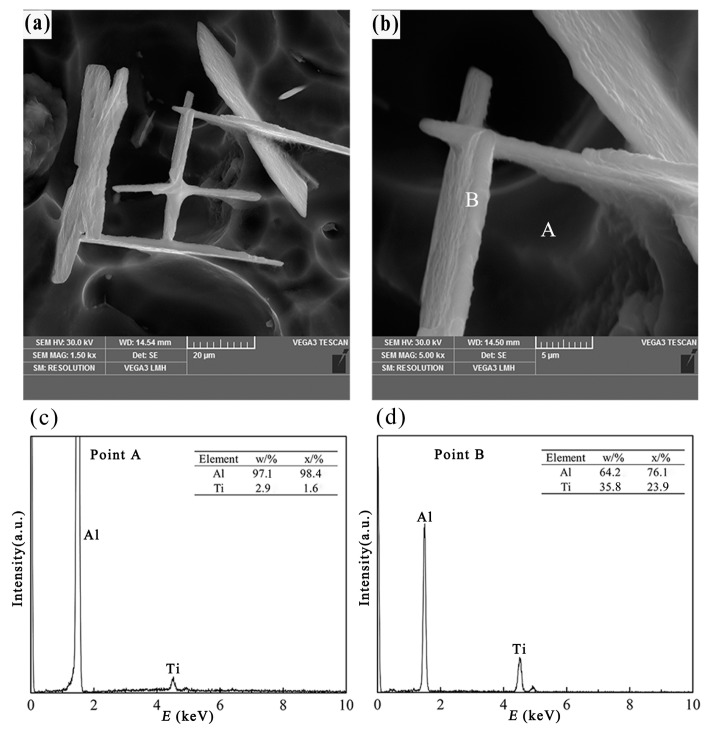
Three-dimensional morphology and point scan elements analysis of flake-like TiAl_3_, (**a**,**b**) three-dimensional morphology of the flaky TiAl_3_ particle; (**c**) EDS spectra of point A; (**d**) EDS spectra of point B.

Figure 8a,b shows the three-dimensional morphology of flake-like TiAl_3_ particles in Al–4Ti master alloys, and the melt was solidified in a sand mold. When the cooling rate is low (about 0.31 K/s, at a distance of 20 mm from the bottom), the morphology of the TiAl_3_ particles is different from that in Figure 6 and Figure 7. The primary particles appear as thin flaky or plate-like shapes when the melt was cooled by sand, and similarly shaped particles have been observed in a rapidly solidified Al–Ti and Al–Ti–C master alloy, as reported in References [20,21,22]. The existence of TiAl_3_ particles was proved by point scan analysis in Figure 8c,d. It can be seen from Figure 8a that the thin flaky TiAl_3_ particles were aggregating together with a certain order. It is clearly shown that the thin flaky TiAl_3_ particles were crossed or perpendicular with each other. However, the TiAl_3_ particles still showed a small and thin flaky morphology, finally. It can be speculated that the second phase in Figure 8a,b was the origin of the morphology of flaky TiAl_3_ particles, which were approximately 50 μm in length, 25 μm in width, and 2 μm in height. Maybe, due to the effect of cooling rate and crystallographic orientation, the flaky TiAl_3_ particles were distributed uniformly in the initial solidification. Because of the extension of cooling time and the change of the alloy components, the tiny, flaky TiAl_3_ particles grew up and dropped off from the matrix, as shown in Figure 8a. In addition, Figure 8b reveals that the volume/surface area ratio of the flaky TiAl_3_ particulates was smaller than that of the blocky ones, indicating that the strain energy has a great impact on the growth of these particles.

When the cooling rate is 0.31 K/s, it means that the cooling time is extended and the Ti atoms stay in the Al melt for a long time. During this time, the TiAl_3_ particles are formed by the reaction of titanium and aluminum. It needs to be pointed out that the growth of TiAl_3_ is controlled by the long-range diffusion of Ti atoms, which was described by Arnberg et al. [18]. When the cooling rate is low, the TiAl_3_ particles are almost flake-like. It reveals that the growth rate in the direction that is parallel to the flake is larger than that in the vertical direction. This is in agreement with the study of Wang et al. [16] and Arnberg et al. [18], who suggested that the growth rate is restricted in the [001] direction and the growth rate in [110], [100], and [010] directions is larger than that in [001] direction. It is well known that the atomic density of a crystal plane is inversely proportional to the growth rate. According to the tetragonal crystal structure of TiAl_3_ (a = 0.3848 nm and c = 0.8596 nm) in the references [16] and [18], it can be calculated that the atomic density on (001) plane (k = 1/a^2^ = 6.75) is larger than that on (100) and (010) planes (k = 2/(ac) = 6.05). So, the reason of the growth rate is suppressed in the [001] direction can be explained. In addition, the low cooling rate results in a high-temperature environment for a long time, in which is hard to form a supersaturated solid solution of Ti in aluminum. Therefore, the thin flaky TiAl_3_ particles are produced at a low cooling rate.

## 4. Conclusions

1. Different morphologies of TiAl_3_ particles in Al–4Ti master alloy could be acquired by adopting different cooling rates. The petal-like, blocky, and flake-like TiAl_3_ particles were respectively acquired at the cooling rates of 3.36 K/s, 2.57 K/s, and 0.31 K/s.
2. With the decrease of cooling rate, the morphology of TiAl_3_ particles in the prepared master alloy changed from petal-like to blocky and, finally, to flake-like. It was also found that the petal-like TiAl_3_ particles could be formed by thick plates during the solidification.
3. The three main types of TiAl_3_ particles did not differ in composition. Moreover, the phase inversion temperature of Al–4Ti master alloy did not change with different particles morphologies.
